# Strontium leaching from municipal waste subjected to incineration

**DOI:** 10.1007/s10653-024-01998-1

**Published:** 2024-06-07

**Authors:** Kicińska Alicja, Caba Grzegorz

**Affiliations:** Department of Environmental Protection, Faculty of Geology, Geophysics and Environmental Protection, AGH University of Kraków, Mickiewicza 30 Av., 30-059 Kraków, Poland

**Keywords:** Leachates, Fraction of municipal wastes, Environmental risk assessment

## Abstract

The aim of the study was to determine the content and leachability of Sr in ashes obtained through combusting municipal waste in household furnaces. The waste had been collected as a mixed stream and as separate fractions (i.e. furniture, sponges, waste paper, PCV packaging, plastic-coated paper cartons, imitation leather, rubber, textiles and polystyrene). Using single-step chemical extractions, (HCl + HNO_3_, H_2_O, 0.01 M CaCl_2_, 0.1 M CH_3_COOH), we determined the total content of Sr (TC) and proportions of the following fractions: water-leachable, phytoavailable and easily soluble and bound to carbonates. We also analyzed the effect of reducing pH in the extraction solutions on St leachability from the study material. The study showed that Sr concentration in ash generated from the combustion of conventional fuels, alternative fuels and municipal waste ranged from 114 to 1006 mg/kg. The largest amounts of Sr were found in ash generated from the combustion of alternative fuels (coal pellets 488–1006 mg/kg), conventional fuels (hard coal 430–670 mg/kg) and mixed waste (237–825 mg/kg). The most mobile fraction of Sr (water-leachable) comprised from 1.3% to nearly 91% TC; the phytoavailable fraction and the ion-exchange and carbonate-bound fraction comprised 3–92% TC and 9–72% TC, respectively. We also found that the greatest pH reductions do not always entail the greatest amounts of extracted Sr. A much more significant factor in this respect is the mineral and chemical composition of primary materials, which can buffer changes in pH. The Risk Assessment Code (RAC) values pointed to a varied environmental risk and the highest RAC values (> 70) were found for coal pellets, wood pellets, straw, rubber and plastic containers for mixed oils.

**Supplementary Information:**

The online version contains supplementary material available at 10.1007/s10653-024-01998-1.

## Introduction

Strontium (Sr), an alkaline earth metal, is an element prevalent in nature (Burger & Lichtscheidl, 2019). Its biogeochemical properties are comparable to those of calcium (Ca). In weathering processes, Sr behaves similarly to calcium and transits into the solution in the form of bicarbonate (Gautsch, 1961). The quantitative ratio of Ca/Sr is considered to be an indicator for the assessment of relative concentration of Sr and its toxicity (Copeland et al., 2011; Koarai et al., 2020). It is assumed that the Ca/Sr ratio < 8 is unfavorable to living organisms (Ilyas et al., 2020; Izumisawa et al., 1994; Kabata-Pendias & Pendias, 1999; Kolitz et al., 2021).

The main source of radioactive strontium is nuclear fission (Bogdanovich et al., 1998; Mirzoeva et al., 2022; Tsukada et al., 2023). Out of Sr radioisotopes produced through nuclear fission, ^90^Sr is particularly dangerous to humans, with its amount reaching up to 5.8% (Ageeva et al., 2010). A long half-life of ^90^Sr (i.e. 28.8 years) and its chemical properties similar to those of calcium make radioactive strontium accumulate in the skeletal system, mainly the cancellous bone, having entered the human body via soil–plant-animal or soil–plant-food cycles (Minami & Suzuki, 2018). This form of Sr is easily absorbed by plants from soil and rapidly incorporated into the food chain, which entails a substantial environmental risk (Chatterjee et al., 2020; Ehlken & Kirchner, 2002; Gupta et al., 2018; Huang et al., 2010). ^90^Sr isotope is relatively mobile, especially in light acidic soils. It remains in the stratosphere for 2–10 years and then moves to the troposphere (Kabata-Pendias & Mukherjee, 2007). Along with radioactive fallout, it is deposited on plants, soil and water (Koshikawa et al., 2016). In soil, strontium is mainly (about 70%) sorbed in the upper horizons (i.e. at the depth of 0–5 cm BGL). Depending on the sorptive properties of different soil types, sorption processes occur at a varying intensity (the greatest has been reported for clays and peats). Sr content in acidic soils is usually lower than in soils containing calcium carbonate and increases in deeper levels of the soil profile (Wang et al., 2012; Willey, 2014). Strontium is fairly easily sorbed by clay minerals and organisms forming skeletons out of calcium. Consequently, its content increases in clayey sedimentary rocks and carbonate formations (Liu et al., 2012; Wenzel et al., 1999). Furthermore, organic matter, e.g. peat, also displays a high sorption capacity in relation to this metal (Kabata-Pendias & Pendias, 1999). In light acidic soils, strontium is easily leached down the soil profile and in calcareous soils, it is readily activated, as it is substituted by other monovalent and divalent cations (Wang et al., 2017). The content of this element in the upper horizons of soil ranges between 5 and 1000 mg/kg. The mean content of Sr (mg/kg) in various soils is as follows: podzolic—87, brown—210, sandy—10, loamy—24, with the anthropogenic enrichment factor being relatively high (Kabata-Pendias & Pendias, 1999). In soils adjacent to industrial areas, the reported Sr content is substantially higher, e.g. in the Lubin-Głogów Copper Belt (300 mg/kg) or Upper Silesia—a region of hard coal mining and metal industry (600 mg/kg). Another source of strontium in soils are fertilizers, which contain significant amounts of this element e.g.: phosphate fertilizers 25–500 mg/kg, calcium fertilizers 610 mg/kg or manure 80 mg/kg (Douglas et al., 2012).

Plants absorb strontium from soils in varying degree, depending on the type, layer or properties of soil (Qi et al., 2015; Russell & Garner, 1959; Vose & Koontz, 1959). The soil content of Sr also largely depends on its concentration in the bedrock (Okamoto et al., 2008; Sasmaz & Sasmaz, 2017). Sr concentrations in magmatic rocks have been reported at 2–600 ppm, in sedimentary rocks at 20–600 ppm and in coals at about 300 ppm (Kabata-Pendias & Pendias, 1999). High Sr levels are found in crystalline celestine (SrSO_4_) clusters, which occur in sulfur deposits.

The use of strontium in industry is not extensive, yet quite common. It is used in non-ferrous metallurgy, the ceramics and glass industries, the manufacture of paints, pyrotechnics and in the pharmaceutical industry. The main source of Sr contamination worldwide is coal combustion, whereas locally, it is sulfur mining (Kabata-Pendias & Pendias, 1999; Sasmaz et al., 2021). There are numerous publications (Longjun et al., 2008; Pathak, 2017; Weiyong & Diping, 1997) on Sr content in industrial wastewater (mean Sr content: 276 mg/kg) and municipal wastewater (40–360 mg/kg d.m.).

The increasing volume of municipal waste necessitates a search for ways to reduce this amount (e.g. by incineration) and possibilities of reusing waste (e.g. to remediate of fertilize soil). However, in order for ashes to be used in this way, they must meet appropriate standards aimed mainly at protecting the soil environment, including crop quality and so-called soil health (Prunier et al., 2015). In order to determine the environmental safety associated with the release of potentially toxic elements from a variety of materials (including ash), extractions with leaching solutions are used (e.g. with strong inorganic and organic acids, oxalates, ammonium acetate etc.). This process allows to identify residual fractions and fractions that are easily or sparingly soluble in an acidic or alkaline environment or those bound to Fe or Mn hydroxides (Kabata-Pendias & Pendias, 1999).

In light of the data presented above, the present study aims to analyze the occurrence and mobility of Sr in different fractions of municipal waste subjected to thermal treatment. The novel aspect to this study involves: (i) measuring Sr content in municipal waste, both in the mixed waste stream as well as in separately collected fractions combusted in household furnaces, (ii) calculating the degree of water leaching of Sr from ash generated by the combustion of this waste and (iii) determining the environmental risk indicator (RAC) stemming from the penetration of easily leached Sr fraction from inadequately secured ash into soils and ground water.

## Research materials

### Material preparation

The first stage of the study involved selection, collection and preparation of fuel material most commonly used in individual households. These were samples of (Table 1):

- Conventional fuels (CF, *n* = 3) comprising hard coal from various fuel storage sites,
- Alternative fuels (AF, *n* = 8) including: coal pellets, wood pellets, straw and green waste,
- Alternative fuels—wood (AF_W_, *n* = 5) including the wood of walnut *Juglans* L., willow *Salix* L., acacia *Acacia* Mill. and oak *Quercus* L.,
- Mixed municipal waste (MMW, *n* = 3), comprising mixed samples of all waste fractions generated in households,
- Municipal waste (MW, *n* = 11), comprising the following separately collected fractions: paper, textiles, plywood, upholstery foam, artificial leather, rubber, polystyrene and PVC packaging (used to store mineral oils or plant protection agents).

**Table 1:** Total concentration (TC) of Sr in conventional and alternative fules and municipal wastes

Group of ashes	Primary burned material	Total C ± SD	Min.–Max	Av
	Coal I	670.2 ± 2.7		
CF	Coal II	430.1 ± 1.4	430.1**–**670.2	528 ± 102
	Coal III	484.8 ± 4.5		
	Coal pellets (ekogroszek) I	488.6 ± 3.5		
	Coal pellets (ekogroszek) II	1006.3 ± 4.0		
	Coal pellets (ekogroszek) III	633.1 ± 2.6		
	Wood pellets I	349.3 ± 4.3		
AF	Wood pellets II	453.0 ± 0.5	165.9**–**1006.3	430 ± 270
	Leaves of tree	176.7 ± 1.2		
	Straw	169.8 ± 1.2		
	Green waste	165.9 ± 1.2		
	Wood of nut	338.7 ± 1.3		
	Wood of wet willow	333.5 ± 4.3		
AFw	Wood of dry willow	370.0 ± 2.4	298.6**–**440.6	356 ± 48
	Wood of acacia	440.6 ± 1.4		
	Wood of oak	298.6 ± 0.3		
	Mixed municipal wastes I	237.1 ± 0.2		
MMW	Mixed municipal wastes II	283.7 ± 3.3	237.1**–**825.4	449 ± 267
	Mixed municipal wastes III	825.4 ± 2.8		
	Plywood	369.6 ± 1.4		
	Sponges	113.9 ± 0.1		
	Waste paper	254.6 ± 1.3		
	Plastic-coated paper cartons	306.2 ± 3.3		
MW	PCV packaginig (mineral oil)	331.7 ± 0.3		
	PCV packaging (mix oil)	145.9 ± 0.1	113.9**–**516.4	315 ± 115
	PCV packaging (plant protection prod.)	370.5 ± 1.4		
	Imitation leather	443.8 ± 2.4		
	Rubber	220.0 ± 0.2		
	Textiles	516.4 ± 2.5		
	Polystyrene	414.6 ± 1.4		
*For all samples*			113.9–1006.3	387 ± 194
CRM	Reference values Measured values (AO%) LOD LOQ	264 ± 13.9 238 ± 11.6 (*90.15*) 0.0358 0.1552		

*CF* conventional fuels, *AF* alternative fuels, *AFw* alternative fuels (wood), *MW* municipal wastes, *MMW* mixed municipal wastes, *CRM* certificate material, *LOD* limit of detection, *LOQ* limit of quantification

The primary material, about 5 kg per each sample, was collected between November 2022 and March 2023. Next, the material was fragmented (about 2–5 cm fragments), homogenized, reduced and divided into furnace feed portions, about 0.05 m^3^ each.

The CF samples represented 3 lump grades: hard coal no. I (from a coal storage site in Małopolska, Poland, EU) was coarse, with lump size of 10–20 cm, hard coal no. II (bought in a storage site in Mazowieckie Voivodeship, Poland, EU) was finer, with lump size of about 5–10 cm, and hard coal no. III (from Podkarpacie, Poland, EU) was the finest, resembling coal pellets, with lump size of about 2–5 cm.

The AF samples were processed materials, which had previously been fragmented. These were: baled straw dried for 1 year with a low level of moisture (about 30%) and green waste with a multi-species felling composition. Coal pellets had a high calorific value, exceeding 28.5 MJ/kg, and a low level of moisture of 4–7%. They were sourced from hard coal mines called “Wujek”, “Wieczorek” and “Mysłowice” located in the Silesian Coal Basin (Poland). Wood pellets were fine-grained (6–10 mm) and had a low level of moisture (3.6–3.9%). They were obtained from pine wood (pellets I) and beech-oak wood (pellets II).

AF_W_ used in the experiment comprised freshly cut (undried) wood obtained as chopped logs from a local logging site.

Municipal waste represented a dry fraction, and was collected in a separate container. All waste samples were processed by fragmenting or crushing.

A single-shaft shredder was used to shred the collected municipal waste, and a laboratory jaw crusher was used for some of the hard materials (i.e. coal and hard waste fractions: furniture board) (Makrum type L44.41).

### Ash sample preparation

**Fig. 1:**
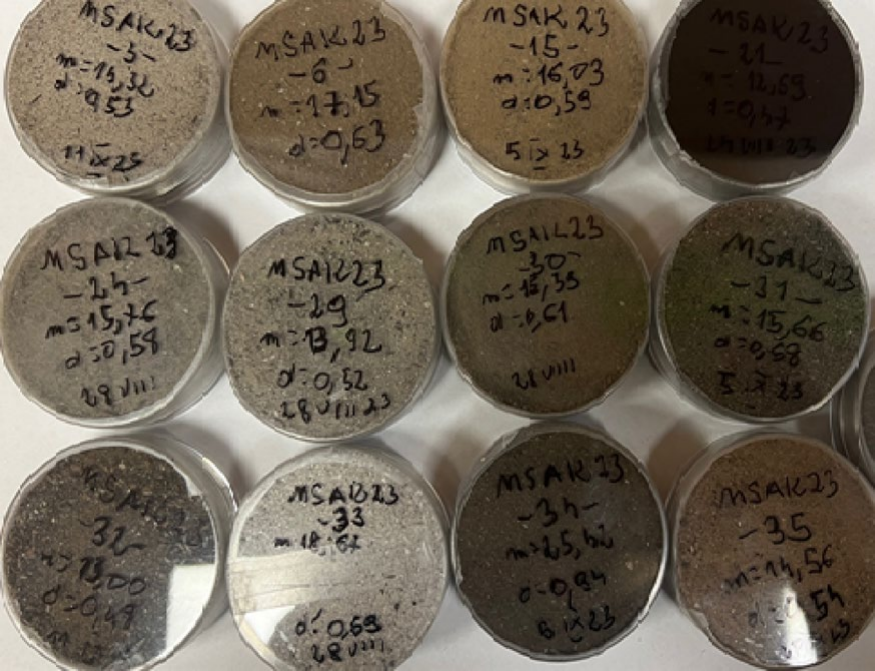
Research materials—ashes

The second stage involved combusting the material collected. The primary materials were incinerated in a Keller 10 kW chamber furnace (with nominal heating capacity of 10 kW, efficiency of 80%, and maximum working pressure of 0.25 MPa). Each of the primary materials yielded a portion of ash from which the study material was selected. The crucial aspect was to maintain similar combustion conditions and clean the furnace chamber before burning the next portion of fuel. Using the above methods, 30 ash samples were obtained, with the weight of about 1 kg each. We observed that the ash samples differed in color (Fig. 1). A morphological description of the ashes obtained has been described in a paper by Kicińska and Caba (2021).

The exact procedure of ash sample preparation has been described by Kicińska et al. (2022a).

## Research methods

During the first stage of the laboratory work, all the samples were air-dried and separated into < 2 mm and ≥ 2 mm fractions. Next, the following parameters were determined for the < 2 mm fraction:

- Total Sr and Ca content using a mix of concentrated acids (38% HCl + 65% HNO_3_ at 3:1 ratio) in a mineralizer at 130 °C (USEPA 1996, EPA 3050B);
- Share of water-leachable fraction of Sr (at 1:10 solid phase/solution ratio) (PN-EN 12457-2:^2006^);
- Share of phytoavailable fraction of Sr, using a single-step 0.01 M CaCl_2_ extraction (at 1:10 solid phase/solution ratio) (Kicińska et al., 2022a, 2022b);
- Share of exchangeable fraction of Sr—easily soluble in an acidic environment, containing metals at the exchange positions as well as bound to carbonates; extraction with 0.1 M CH_3_COOH (at 1:10 solid phase/solution ratio) (Quevauviller, 2003);
- Sr mobility in gradually acidified solutions, first stage—no acid added; second stage—0.5 cm^3^ 1 M HNO_3_; third stage—1 cm^3^ 1 M HNO_3_ (1:10 solid phase/solution ratio) (Kicińska et al., 2022a, 2022b);

The environmental risk related to the presence of Sr in ashes from household furnaces was calculated using the Risk Assessment Code (RAC). The method involves comparing the percentage of cations found in ion-exchange positions and bound to carbonates, with their total concentration in a given sample. RAC was calculated with the following formula no. (1):

1 $$RAC = \frac{amount\;of\;cations\; of\; each\; element\; bound\; with\; ion\; positions\; and\; carbonates}{{total \;content \;of\; element}} \times 100\%$$

Values between 1 and 10% denoted low risk, 11–30%—medium risk, 31–50%—high risk, and over 50%—very high risk (Håkanson, 1980).

Total Sr and Ca concentrations in post-extraction solutions were determined with an ICP-MS instrument in an accredited hydrogeochemical laboratory (certificate of accreditation PCA no. AB1050) at AGH University of Science and Technology in Kraków. Precision of the measurements was 10% and accuracy ranged from 95 to 105%. DL of the instrument used was 3∙10^–4^ mg/dm^3^. The control system of the analyses (QA/QC) followed the standard PN-EN ISO 17294-2:2016-11 using blank samples, doubled samples (min. 25%) and marked samples in each series of determinations. Also the Standard Reference Material (CRM048, lot: LRAB1604) was analyzed, for which the differences of the Sr determinations did not exceed 10% (Table 1). The results were statistically compiled using Excel 2013 and Statistica *ver*.13.3 software.

## Results and discussion

### Total Sr content in ash

The total concentration of Sr in ash generated from the combustion of conventional fuels, alternative fuels and municipal waste ranged widely from 114 to 1006 mg/kg (Table 1). The largest amounts of Sr were found in ash generated from alternative fuels (coal pellets 488–1006 mg/kg), conventional fuels (hard coal 430–670 mg/kg) and mixed waste (237–825 mg/kg). High concentrations of Sr were observed in ash obtained from the combustion of acacia wood (440 mg/kg), imitation leather (423 mg/kg) and polystyrene (414 mg/kg).

A comparison of individual material groups showed that the mean Sr concentrations decreased in the following order (data in mg/kg): hard coal (528) > mixed municipal waste (449) > alternative fuels (430) > wood (356) > selected fractions of municipal waste (315).

The analysis showed that separate collection of municipal waste is of great pro-environmental importance due to the varying total Sr content in individual fractions. The highest amount of Sr was found in textiles (516 mg/kg), slightly lower in artificial leather and polystyrene (444–414 mg/kg), substantially lower in PVC packaging (370–332 mg/kg) and the lowest in upholstery foam and rubber (114 and 220 mg/kg, respectively). Municipal waste, collected from single-family homes located in rural areas, displayed a considerably higher Sr content (237–284 mg Sr/kg). Finally, one of the highest amounts of Sr (835 mg/kg) was found in mixed waste from multi-family houses located in a large city. This fact may be due to the specific conditions in the country where this study was conducted (Poland, EU). The amount of waste generated per capita in Poland between 2011 and 2020 ranged between 319 and 346 kg, with the amount of waste collected in urban areas being approximately 2.7 times higher than that in rural areas (ec.europa.eu). In Western European countries, such as Germany, Denmark or the Netherlands, the amount of municipal waste collected per capita is approximately two times higher (626–641 kg). This does not point to lower amounts of generated waste, but rather shows that in rural areas some municipal waste is combusted in household furnaces. Furthermore, it is a fairly common practice in rural areas to dump incineration residues (ash) on arable soils, which in the case of high levels of potentially toxic elements, including Sr, can pose a significant environmental risk, not only to soils. Sr content in the upper horizons of soil is reported to range from 5 to 1000 mg/kg, with sandy soils containing an average of 44 mg/kg (Poland), through 118 mg/kg (Australia) to 125 mg/kg (USA). In silt and clay soils, the content is considerably higher, reaching about 300 mg/kg (Kabata-Pendias & Mukherjee, 2007; Prunier et al., 2015). The amount of Sr in industrial wastewater is 270 mg/kg, whereas in municipal wastewater, it is markedly lower, namely 75 mg/kg (Kabata-Pendias & Pendias, 1999; Zhou et al., 2016).

When comparing the values obtained to literature data, it is difficult to find studies focusing on ash from household furnaces. Spivak-Birndorf et al. (2012) analyzed Sr content in coal utilization by-products, namely fly ash (FA), bottom ash (BA) and flue-gas desulfurization product (FGD). Sr concentrations reported by these authors were similar to our results (mg/kg): 235–2946, 482 and 321, respectively, but their study material was sourced from an industrial energy facility.

### Water-leachable fraction

The concentrations of Sr in leachates of ash ranged from 0.08 to 6.09 mg/dm^3^, which equaled 4.31–302.37 mg/kg by weight (Table 2). The highest amounts of the element (302 mg/kg) were leached from ash obtained through combusting mixed waste collected from multi-family houses located in a large city. A considerable amount of Sr was found in leachates of ash generated from burning hard coal (90–251 mg/kg), rubber (221 mg/kg) and artificial leather (216 mg/kg).

**Table 2:** Water-leaching (WL) of Sr from conventional and alternative fules and municipal wastes

Group of ashes	Primary burned material	Sr		Av	% of TC
		(mg/L)	(mg/kg)		
	Coal I	3.6	180.7		27
CF	Coal II	1.8	90.8	174 ± 66	21
	Coal III	4.9	251.9		52
	Coal pellets (ekogroszek) I	2.2	109.7		22
	Coal pellets (ekogroszek) II	1.8	91.1		9
	Coal pellets (ekogroszek) III	0.2	8.3		1
	Wood pellets I	2.6	130.0	62 ± 50	37
AF	Wood pellets II	2.2	110.7		24
	Leaves of tree	0.2	8.2		5
	Straw	0.1	4.3		3
	Green waste	0.6	31.8		19
	Wood of nut	0.1	6.1		2
	Wood of wet willow	0.3	12.6		4
AFw	Wood of dry willow	0.8	38.9	35 ± 23	15
	Wood of acacia	1.3	65.4		18
	Wood of oak	1.1	53.6		11
	Mixed municipal wastes I	1.1	53.6		23
MMW	Mixed municipal wastes II	2.7	136.4	164 ± 103	48
	Mixed municipal wastes III	6.1	302.4		37
	Plywood	0.3	16.7		5
	Sponges	0.4	20.2		18
	Waste paper	1.1	59.6		23
	Plastic-coated paper cartons	2.3	120.8		39
MW	PCV packaginig (mineral oil)	0.5	24.7	104 ± 67	7
	PCV packaging (mix oil)	1.3	66.1		45
	PCV packaging (plant protection prod.)	2.0	96.9		26
	Imitation leather	4.3	216.5		51
	Rubber	4.9	220.7		91
	Textiles	2.9	143.0		28
	Polystyrene	2.8	141.9		34
For all samples			94 ± 78		25

Aberration under Table 1

A comparison of individual material groups showed that the mean Sr concentration decreased in a slightly different order than in the case of the total content of this element (data in mg/kg): hard coal (174) ≅ mixed municipal waste (164) > selected fractions of municipal waste (104) >  > alternative fuels (62) >  > wood (35). This points to a fivefold difference between materials with the highest and the lowest mean Sr leachability (hard coal vs. different types of wood). Markedly high St concentrations were observed in the AF group. Over 100 mg Sr per kg was leached into water extracts of ash generated by combusting wood pellets and coal pellets. A similar situation was observed in the case of textiles, polystyrene and PVC packaging.

The water-leachable fraction is of particular environmental importance because, firstly, it is most mobile and easily incorporated into biogeochemical cycles and, secondly, it is readily absorbed by organisms and penetrates into the groundwater. When analyzing the obtained results in the context of this type of environmental risk (Kicińska, 2019b), we found that:

- The highest share of this fraction (over 50% of the total concentration; TC) was observed in some types of hard coal, artificial leather and rubber (over 90%);
- A considerable share of this fraction (30–50% TC) was observed in mixed municipal waste, PVC packaging and polystyrene;
- A moderate share of this fraction (20–30% TC) was observed in hard coal, wood pellets, waste paper and textiles.

Given the poor solubility of Sr salts (these occur mainly in the form of carbonates and sulfates), the amounts identified in the present study are in line with the concentrations found in river water, which amount to 0.03–5 mg/dm^3^ (Shao et al., 2018).

### Sr mobility

**Fig. 2:**
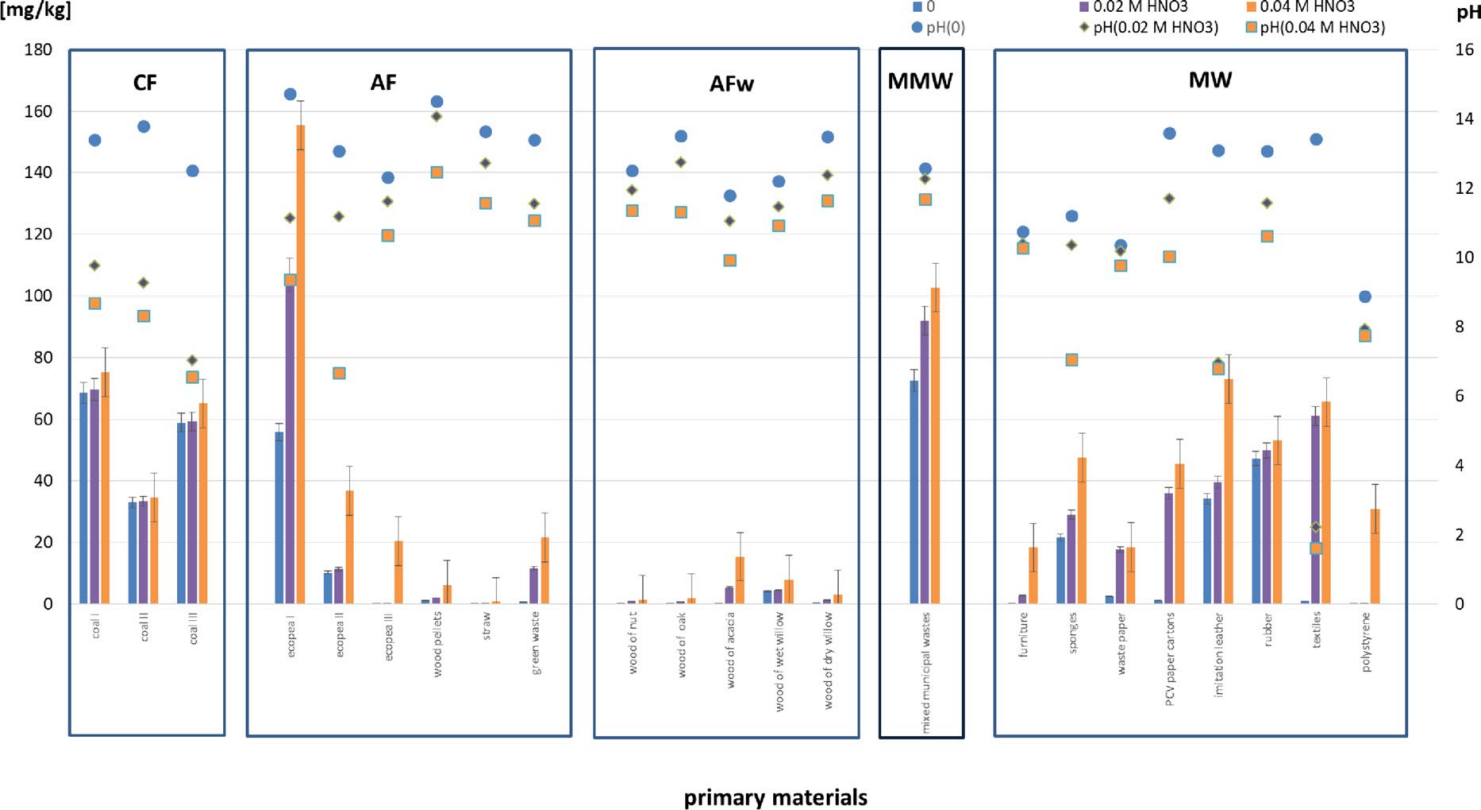
Leaching of Sr [mg/kg] from ashes in gradually acidified solutions

The mobility of elements in changing environmental conditions has been the subject of numerous scientific studies (e.g. Kicińska & Caba, 2021; Kicińska et al., 2022a, 2022b; Wasserman et al., 2024). The prevalent relationship that exists between soil pH and element solubility prompted us to perform an experiment that would allow us to determine the amount of Sr leached from ash subjected to gradual acidification (Fig. 2).

The pH of the water extracts of ash was definitely alkaline, ranging between 8.88 (polystyrene) and 14.73 (coal pellets) (Table S1). In the first stage of acidification (0.02 M HNO_3_), the pH of all the solutions decreased (ΔpH = 0.18–11.20), while Sr mobility increased. The greatest pH decrease was observed for: hard coal (ΔpH = 3.63–5.48) and two fractions of municipal waste, namely artificial leather (ΔpH = 6.13) and textiles (ΔpH = 11.20). A double dose of acid (0.04 M HNO_3_) did not result in any dramatic pH reduction. Considerable changes were observed only in the case of two samples, (coal pellets II) and upholstery foam, with Δ pH of 4.52 and 3.30, respectively. As for the other samples, Δ pH was < 1.5, which points to a very good buffer capacity associated with acidification resistance. The exception was ash generated by combusting textiles. Here, the pH of the solutions was either neutral or alkaline (Fig. 2).

When analyzing the amounts of total Sr extracted through gradual acidification, we found that despite the greatest pH decrease in hard coal, there was no significant increase in Sr mobility.

The same applied to artificial leather. However, even though the first acid dose did not cause any significant decrease in pH, Sr mobility increased:

- Over 10 times in the case of ash obtained from green waste (1620%) and plywood (1881%) combustion,
- Over 20 times in the case of ash obtained from acacia wood (2008%) and PVC packaging (2973%) combustion,
- 60 times in the case of ash obtained from textile (5915%) combustion.

A double dose of acid added to the ash solutions led to an increase in Sr mobility in the case of acacia wood (4012% as compared to non-acidified solutions), plywood (over 10,573%) and polystyrene (27,180%). In one sample of ash obtained from the combustion of coal pellets (coal pellets III), the amount of extracted Sr increased 300 times.

The highest amounts of Sr were extracted in the second acidification stage from coal pellets I (155 mg/kg) and mixed municipal waste (103 mg/kg), despite high pH (9.36 and 11.68, respectively).

These results point to an untypical relationship, namely that the greatest pH reduction does not always entail the greatest amount of extracted Sr (Wasserman et al., 2024). A much more significant factor is, as shown by Choi et al. (2023) and Sysoeva et al. (2005), the mineral and chemical composition of primary materials, which can buffer changes in pH. Other important factors affecting radionuclide mobility in soil during migration processes, as mentioned by Gupta et al. (2018), are soil texture, exchangeable calcium and potassium content and organic matter content. Studies by Douglas et al. (2012), and Wasserman et al. (2024) showed that Sr is readily leached down the profile in acidic soils, whereas in calcareous soils, it is easily activated due to replacement by other divalent cations.

Changes in soil pH (i.e. alkalization or acidification) alter chemical activity of Sr by breaking bonds between Sr ions and organic and inorganic compounds, which consequently changes the form of Sr in a given matrix. Substances present in ash (mainly carbonates) determine its high (alkaline) pH. However, upon entering the environment, ash undergoes further transformations, and the substances present in soil and water may act as extractants, causing meatal ions to become more or less active, or increasing or decreasing the environmental risk related to their presence.

### Sr phytoavailability

We studied Sr phytoavailability because we considered the possibility of the ash being used as a fertilizer and thus we wanted to assess the risk it posed when deposited on arable land. Literature data show that plants absorb strontium from soils in varying degree, depending on the plant species and part, and the properties of soil in which they grow (Sysoeva et al., 2005; Techer et al., 2017). The effect of environmental processes on the absorption of radioactive trace elements by plant roots and the variability of the transfer factor (TF) have been extensively described by e.g. Ehlken and Kirchner (2002), and Guillen (2018).

**Fig. 3:**
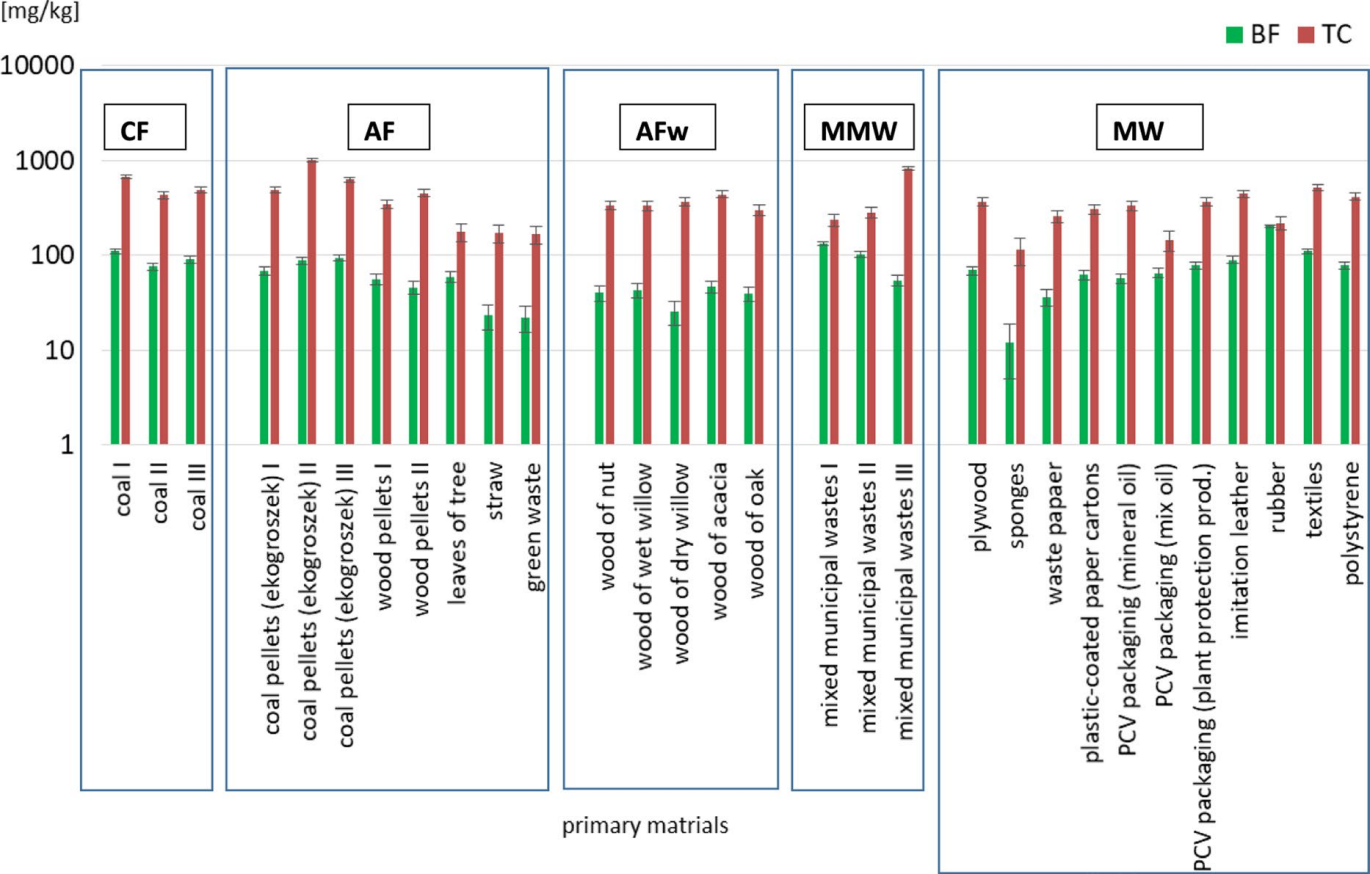
Total content (TC) and phytoavailability (BF) of Sr in ashes

The bioavailable or phytoavailable fraction forms only a part of the total concentration and its proportion is determined by means of extraction with various reagents, including 0.05 M EDTA, 1 M HCl or 0.01 M CaCl_2_. In the material studied, the share of this fraction ranged from 11.98 mg/kg (spongies) to 202.19 mg/kg (rubber), equaling 7–92% TC (Fig. 3).

The mean share of the phytoavailable fraction, (expressed as a percentage of the total concentration of Sr) in individual material groups decreased in the following order: mixed municipal waste (33) > selected fractions of municipal waste (27) >  > hard coal (18) > alternative fuels (16) > wood (11).

The results showed that Sr in biological material, i.e. wood of different tree species, had the lowest phytoavailability (25.18–46.85 mg/kg), which ranged from 7% TC (dry willow wood) to 13% TC (acacia wood). In turn, the highest bioavailable amounts of Sr were found in mixed municipal waste (53.90–132.66 mg/kg), equaling 7–56% TC (Table 3).

Studies by Kabata-Pendias and Pendias (1999), and Landstetter et al. (2010) demonstrated that Sr content in lucerne and clover exceeded 600 and 200 mg/kg d.m., respectively, whereas in cereals and vegetables, it was markedly lower, namely 1–20 mg/kg and 2–75 mg/kg, respectively.

**Table 3:** Bioavaible fraction (BF) of Sr (mg/kg) in conventional and alternative fules and municipal wastes

Group of ashes	Primary burned material	C [mg/kg]	Min.–Max	Av	% of TC
	Coal I	110.70			17
CF	Coal II	76.42	76.42–110.70	92.44	18
	Coal III	90.19			19
	Coal pellets (ekogroszek) I	68.07			14
	Coal pellets (ekogroszek) II	88.06			9
	Coal pellets (ekogroszek) III	94.26			15
	Wood pellets I	55.62	22.25–94.26	57.08	16
AF	Wood pellets II	45.78			10
	Leaves of tree	59.41			34
	Straw	23.21			14
	Green waste	22.25			13
	Wood of nut	39.94			12
	Wood of wet willow	42.56			13
	Wood of dry willow	25.18			7
AFw	Wood of acacia	46.85	25.18–46.85	38.81	11
	Wood of oak	39.52			13
	Mixed municipal wastes I	132.66			56
MMW	Mixed municipal wastes II	103.31	53.90–132.66	96.62	36
	Mixed municipal wastes III	53.90			7
	Plywood	69.48			19
	Sponges	11.98			11
	Waste papaer	36.19			14
	Plastic-coated paper cartons	62.09			20
MW	PCV packaginig (mineral oil)	56.78			17
	PCV packaging (mix oil)	65.21	11.98–202.19	78.12	45
	PCV packaging (plant protection prod.)	77.89			21
	Imitation leather	89.51			20
	Rubber	202.19			92
	Textiles	110.31			21
	Polystyrene	77.69			19
For all samples			11.98–202.19	69.24	21

Aberration under Tab. 1

### Environmental risk

The aim of environmental risk assessment is to identify the likelihood of adverse changes in the natural environment or long-term effects of these changes stemming from the negative impact of a substance/element on the environment (Kicińska & Mamak, 2017; Kicińska, 2019a; Yankovich et al., 2010). This notion is close to that of ecological risk assessment, which is a similar process determining the likelihood of adverse environmental effects that can or do occur as a result of exposure to one or more stressors (Khaska et al., 2019; Tsukada et al., 2023). In the present study, such a stressor was Sr. Its presence should be considered in the context of not only total concentration (potentially available) but also co-occurring biogeochemical fractions of this element (e.g. bioavailable concentrations bound to carbonates etc.), which can readily undergo biomagnification and pose a real threat to proper development and health of various organisms (Song et al., 2014). An excess of this element has been shown to cause bone deformities. Sr is also involved in calcium metabolism (Kabata-Pendias & Mukherjee, 2007).

To determine the environmental risk, extraction with 0.1 M acetic acid (CH_3_COOH) was performed to measure the ion-exchange and carbonate-bound fraction, which is easily extractable (especially in an acidic environment) and readily incorporated into biogeochemical cycles. The ratio between this value and total concentration forms the basis for determining the Risk Assessment Code (RAC) and subsequently performing a risk assessment according to the classification proposed by Håkanson (1980).

Based on our analysis, we calculated RAC values (Table 4). Their range was very extensive (9–76), which suggested that the environmental risk posed by the material studied was low (in the case of ash generated by plywood combustion), medium (mixed municipal waste, artificial leather and willow wood) and high or very high (the other ashes). The highest RAC values (> 70) were found for the following primary materials: coal pellets I, wood pellets, straw, rubber and plastic containers for mixed oils. Similar data were published by Tsukada et al. (2023), who demonstrated that the content of the exchangeable fraction in soil samples collected in Aomori, Fukushima and Chornobyl can range from 54 to over 70% TC.

**Table 4:** Risk assessment code (RAC) and Ca/Sr factor for conventional, alternative fules and municipal wastes

Group of ashes	Primary burned material	RAC	Risk	Ca/Sr
	Coal I	53	Very high	63
CF	Coal II	28	Medium	201
	Coal III	42	High	87
	Coal pellets (ekogroszek) I	70	Very high	20
	Coal pellets (ekogroszek) II	62	Very high	241
	Coal pellets (ekogroszek) III	34	High	215
	Wood pellets I	30	High	141
AF	Wood pellets II	76	Very high	148
	Leaves of tree	70	Very high	297
	Straw	62	Very high	20
	Green waste	34	High	241
	Wood of nut	45	High	218
	Wood of wet willow	29	Medium	200
	Wood of dry willow	29	Medium	200
AFw	Wood of acacia	39	High	164
	Wood of oak	40	High	173
	Mixed municipal wastes I	14	Medium	75
MMW	Mixed municipal wastes II	13	Medium	68
	Mixed municipal wastes III	15	Medium	98
	Plywood	9	Low	203
	Sponges	48	High	176
	Waste papaer	57	Very high	412
	Plastic-coated paper cartons	55	Very high	224
MW	PCV packaginig (mineral oil)	59	Very high	83
	PCV packaging (mix oil)	72	Very high	111
	PCV packaging (plant protection prod.)	66	Very high	286
	Imitation leather	59	Very high	130
	Rubber	72	Very high	203
	Textiles	48	High	176
	Polystyrene	57	Very high	412
For all samples			High	177

If RAC:

No Risk < 1

Low Risk 1–10.0

Medium Risk 10.01–30

High Risk 30.0–50

Very High Risk > 50

These results may be of concern given the aforementioned use of ash as a fertilizer in rural areas, dumped directly on agricultural fields. This action may have a negative impact on soil and groundwater. The factors and mechanisms determining the uptake and accumulation of Sr by plants, e.g. the properties of soil particles (minerals) and the availability of organic matter, have been described by Gupta et al. (2018). There is also a particular risk associated with the soil–plant transfer of Sr. The element can enter the human food chain via this pathway, either through direct intake or indirect intake related to animal feeding (Guillén, 2018). As Sr is involved in calcium metabolism, we additionally calculated the ratio of total Ca to Sr. In all samples, it ranged between 20 and 412 (Table 4), which suggests that the content of Sr in ash is not likely to be toxic to living organisms.

## Conclusions

The global mining of strontium has remained at a similar level for years. This element is commonly used in industry and its considerable amounts (over 600 mg/kg) have been reported in conventional (coal) and alternative (coal pellets) fuels, as well as in mixed municipal waste collected from single-family households in large cities.

The environmental risk stemming from high total concentrations of Sr (114–1006 mg/kg) found in ash generated in household furnaces by combusting various types of fuel (ranging from conventional to alternative) and municipal waste (mixed and collected separately) is associated with high leachability of Sr, especially in an aquatic environment.

Based on the analyses conducted, we found that:

- High reduction in pH is not always associated with the highest extraction of Sr,
- The most mobile fraction of this element (water-leachable) comprised from 1.3% to nearly 91% TC,
- The phytoavailable fraction of Sr comprised 3–92% TC,
- The ion-exchange and carbonate-bound fraction comprised 9–72% TC.

These results clearly point to a low (*n* = *1*), medium (*n* = *6*) or high (*n* = *9*) and very high (*n* = *14*) environmental risk related to Sr leaching from ash generated by municipal waste combustion. Our findings also emphasize the importance of adequate storing and securing landfills against the potential resuspension of the smallest ash particles by wind and their deposition on soils and plants.

### Supplementary Information

Below is the link to the electronic supplementary material.

Supplementary file1 (DOCX 15 kb)

## Data Availability

No datasets were generated or analysed during the current study.
