# Supplementary material for: Strontium leaching from municipal waste subjected to incineration
Source: Environ Geochem Health. 2024 Jun 7;46(7):220. doi: 10.1007/s10653-024-01998-1 (PMC11161431; doi:10.1007/s10653-024-01998-1)
Supplement: Supplementary file 1 — Supplementary file1 (DOCX 15 kb) [file 10653_2024_1998_MOESM1_ESM.docx]

Table S1. pH of ashes in gradually acidified solutions

| **group of ashes** | **primary burned material** | **pH** | | |
| --- | --- | --- | --- | --- |
|  |  | **(0)** | **(0.02 M HNO_3_)** | **(0.04 M HNO_3_)** |
|  | coal I | 13.39 | 9.76 | 8.69 |
| CF | coal II | 13.79 | 9.28 | 8.31 |
|  | coal III | 12.51 | 7.03 | 6.56 |
|  | coal pellets (ekogroszek) I | 14.73 | 11.15 | 9.36 |
|  | coal pellets (ekogroszek) II | 13.07 | 11.18 | 6.66 |
|  | coal pellets (ekogroszek) III | 12.32 | 11.62 | 10.65 |
|  | wood pellets I | 14.50 | 14.08 | 12.47 |
| AF | wood pellets II | 13.63 | 12.72 | 11.57 |
|  | leaves of tree | 13.40 | 11.55 | 11.08 |
|  | straw | 14.73 | 11.15 | 9.36 |
|  | green waste | 13.07 | 11.18 | 6.66 |
|  | wood of nut | 12.51 | 11.94 | 11.35 |
|  | wood of wet willow | 13.50 | 12.75 | 11.32 |
| AFw | wood of dry willow | 11.80 | 11.05 | 9.92 |
|  | wood of acacia | 12.21 | 11.47 | 10.93 |
|  | wood of oak | 13.49 | 12.38 | 11.63 |
|  | mixed municipal wastes I | 12.57 | 12.26 | 11.68 |
| MMW | mixed municipal wastes II | 13.58 | 12.89 | 12.55 |
|  | mixed municipal wastes III | 11.59 | 11.58 | 10.89 |
|  | plywood | 10.75 | 10.40 | 10.28 |
|  | sponges | 11.20 | 10.35 | 7.05 |
|  | waste paper | 10.36 | 10.18 | 9.76 |
|  | plastic-coated paper cartons | 12.89 | 11.99 | 11.45 |
| MW | PCV packaginig (mineral oil) | 13.59 | 11.70 | 10.04 |
|  | PCV packaging (mix oil) | 12.89 | 11.78 | 11.25 |
|  | PCV packaging (plant protection prod.) | 12.36 | 12.84 | 12.11 |
|  | imitation leather | 13.09 | 6.96 | 6.80 |
|  | rubber | 13.07 | 11.57 | 10.61 |
|  | textiles | 13.42 | 2.22 | 1.62 |
|  | polystyrene | 8.88 | 7.94 | 7.75 |
